# SMART goals setting and biometric changes in obese adults with multimorbidity: Secondary analysis of a randomized controlled trial

**DOI:** 10.1177/2050312119858042

**Published:** 2019-06-24

**Authors:** Paul Y Takahashi, Stephanie M Quigg, Ivana T Croghan, Darrell R Schroeder, Jon O Ebbert

**Affiliations:** 1 Division of Primary Care Internal Medicine, Mayo Clinic, Rochester, MN, USA; 2 Robert and Arlene Kogod Center on Aging, Mayo Clinic, Rochester, MN, USA; 3 Nicotine Dependence Center, Mayo Clinic, Rochester, MN, USA; 4 Division of Biomedical Statistics and Informatics, Mayo Clinic, Rochester, MN, USA

**Keywords:** Blood pressure, diet, multimorbidity, obesity, pedometer, goal setting

## Abstract

**Objectives:**

Clinicians recommend diet and exercise for overweight/obese patients. We conducted a secondary analysis of a randomized controlled clinical trial evaluating goal setting and pedometer use versus usual care on weight, waist circumference, and blood pressure of patients with multiple chronic conditions.

**Methods:**

In this trial, we recruited and randomized patients over 18 years with multiple chronic conditions. There were two groups with an immediate intervention group who received behavioral coaching and a pedometer versus a delayed control who received the intervention after 2 months. We evaluated body weight, waist circumference, and blood pressure as outcomes. We used analysis of covariance to evaluate differences between the intervention and the control groups.

**Results:**

Of 130 patients, mean age was 63.4 years (SD, 17.3). At 2 months, intervention participants lost 0.2 kg versus a 0.1-kg gain in the control participants (*P* = .44). The immediate intervention group had significantly smaller waist circumference change at 2-month follow-up compared to control at −1.6 cm (95% confidence interval = −3.1 to −0.1), which was driven by an increase in waist circumference in the delayed control group. No difference in systolic blood pressure was observed.

**Discussion:**

We observed no difference in weight or blood pressure between the groups with obesity and multiple chronic conditions.

## Background

Approximately 40% of US adults over age 20 are obese,^1,2^ and obese adults are at risk of adverse health outcomes, including death,^3^ vascular disease like coronary artery disease,^4^ heart failure,^5^ and stroke.^6–8^ Weight loss is an important component of medical care for many obese patients.^9,10^ The most effective way of providing care for obese patients often involves dietary intervention with an exercise program.^9^ The US Preventive Services Task Force recommends behaviorally based methods for weight loss.^11^ SMART goals (specific, meaningful, action-based, realistic, and timely) are one such method to engage patients in weight loss. In a meta-analysis of 18 studies using SMART goals, the authors concluded that SMART goals were effective in reducing weight or improving eating behavior.^12,13^ In a systematic review of nursing-led behavioral methods to reduce weight, 65% of studies showed improvement in weight or body mass index (BMI) using nursing interventions.^14^ While there is good initial evidence on the potential for SMART goals to help with weight loss, there is still uncertainty how well this intervention would work in older, obese patients with multiple chronic conditions (MCC). This higher risk population might benefit from weight loss.

The second component of weight loss involves physical activity. Setting behavioral goals like SMART goals is one method of increasing physical activity.^15^ Technology may be one method to help with behavioral goal setting. In a systematic review of computer-based technologies with or without wearable technology suggested increased activity, reduced weight, and improved biomarkers (like hemoglobin A1C) in patients with behavioral coaching.^16^ Wearable technology (pedometers and accelerometers) may have modest effects on weight loss in diabetic patients.^17^ In a meta-analysis of 26 studies with a younger (49 years) population, there was an association of pedometer use with a BMI decrease of 0.38 kg/m^2^.^18–20^ We note there is a difference in prevalent obesity between men and women,^21^ thus the need to account for sex in the discussion of goal setting. We reported previously on the effect of SMART goal setting and pedometer use on physical activity as measured by step count in a population of overweight or obese adults. We did not observe an increase in step count or markers of physical activity (gait speed and grip strength).^22^ In the original study, we did not report on weight or other biometric outcomes. After a review of the evidence and our previous randomized trial, it is still not clear how behavioral goal setting using SMART goals and pedometers affects weight and other biometric outcomes in a population that is obese with MCC. A novel feature of our study is examining these combined interventions in this population. Our aim was to determine the relationship between SMART goals and pedometer use and patient biometrics such as weight, waist circumference, and blood pressure in patients with intervention compared to a control group. To answer this question, we performed secondary analysis of the randomized controlled trial of the intervention of SMART goal setting and pedometer use versus usual care in adults who were overweight/obese and had MCC.^22^ The primary outcome of interest in this study was weight loss, and secondary outcomes were blood pressure^19^ and waist circumference (abdominal obesity) reduction.^19,20^

## Methods

### Aim, trial design, and setting

The present study analyzed secondary data from a previously reported randomized controlled trial.^22^ The current study uses a randomized controlled trial with immediate entry and delayed entry as the comparison group in a 1:1 ratio. The aim of the study was to evaluate the effect of SMART goals and pedometer use on weight, waist circumference, and systolic blood pressure. The combination of pedometer use and monthly goal setting was the intervention (termed *intervention* hereafter). The trial was performed at a single medical center and was conducted from 1 May 2013 through 9 September 2015. There was no change to the trial after initiation. Patients provided written informed consent for enrollment in the trial. The randomization scheme was developed using computer randomization prior to study enrollment by the statistical team. We utilized block randomization of four, and the results were placed in sealed envelopes. Participants were enrolled by the study coordinator using the randomization mechanism. There was no blinding because of the active intervention. The study was approved by the Mayo Clinic Institutional Review Board and was registered in ClinicalTrials.gov.

### Participants

The inclusion criteria for participants included age ⩾18 years, living within the community, and receiving primary care in the community. Participants were either overweight (BMI, 25.0–29.9 kg/m^2^) or obese (BMI > 30.0 kg/m^2^).^23^ Before the screening, the electronic health records (EHRs) of potential participants were evaluated for potential study eligibility.

Participants were assessed for MCC. The presence of MCC was determined using medical tiering,^24^ which counted the conditions that participants had before screening. The diagnostic *International Classification of Diseases, Ninth Edition*, billing codes were counted, and the participants were placed into 1 of 5 categories from 0 (no chronic conditions) to tier 3 (7–9 conditions) and tier 4 (⩾10 conditions). Overweight/obese participants in tier 3 or tier 4 were eligible (⩾7 conditions). Participants were excluded if they refused EHR review.^25^ Patients also were excluded if they had moderate depression with a Patient Health Questionnaire 9 (PHQ-9) score >10.^26^

The initial eligible pool of potential participants was generated from patients receiving primary care at Mayo Clinic and who met eligibility criteria for age, BMI, and MCC. Potentially eligible patients were recruited by letter. Potential participants were asked to call back to the study team for further verification of eligibility criteria. The team evaluated eligibility criteria using the medical record prior to in-person visit. The study team performed a face to face baseline visit to further verify eligibility criteria and to obtain signed informed consent.^22^

### Immediate intervention

The intervention had two components: goal setting and pedometer use. Goal setting consisted of participants setting SMART goals with the assistance of the study coordinator and written materials.^27^ The length of the sessions was not recorded; however, in general the initial sessions were 1.5 h and the monthly follow-up sessions were 0.5 h. Participants were given nutritional material and a lifestyle modification log book, both available free of charge in all clinic settings, that had instructions about writing down SMART goals. This educational material allowed the participant to document daily food choices, calories, and hunger feelings. The nutritional material emphasized heart-healthy options and stressed proper eating habits and food choices. It also provided information on dietary cholesterol, fat, and sodium and food labels. The study coordinator assisted with primary goal setting that was patient-centered. There was no preset step target or nutritional target. At each monthly follow-up visit, the coordinator reviewed goal setting with the participant. The goals were determined by the patient and could vary from visit to visit. The intensity of the goals was patient driven, and there was no set protocol to develop the goals.

Participants used a pedometer (Omron HJ-112; Omron Healthcare, Inc.) that tracked daily and 7-day step counts. The pedometer has been validated to accurately measure step counts.^28^ Patients were instructed to set their own step goals. They were given an exercise video that described starting an exercise program, with emphasis on 30 min of activity most days of the week. We did not measure adherence or wear time of pedometer use and measured activity through step count only. The study coordinator also provided written exercise literature on starting an exercise program with basic aerobic instruction, stretches, and strength training.^29–32^ Participants enrolled in the immediate intervention group were given the material, and goal setting was discussed at the initial visit and at monthly follow-up visits.

### Delayed control group

Participants in the delayed control group were monitored for outcomes. They had access to the clinical materials on nutrition and activity, which was the same as the immediate intervention group. Controls had a 2-month delay in goal setting following enrollment. After 2 months, these participants were given the goal-setting intervention and had both 1 intervention visit (at month 3) and a final follow-up visit at study completion at 4 months.

### Biometric outcomes

The primary outcome was measured weight loss and weight loss ⩾5%; secondary outcomes were waist circumference and systolic blood pressure. These outcomes are a secondary analysis of an original study and were not developed a priori. The study coordinator measured height of participants when they were not wearing shoes. Weight was measured with an electronic scale and without shoes. BMI was determined from the height and weight. Participants were further categorized into those with ⩾5% weight loss over the duration of the study and those with <5% weight loss. A weight loss >5% has a clinically significant positive health effect, such as insulin sensitivity.^33^ Waist circumference was measured at 2.54 cm above the belly button. The study coordinator measured blood pressure using an automatic blood pressure monitor (Omron Intellisense (Omron Healthcare, Inc.) or Microlife (Microlife Corp)) for one or two readings and recorded blood pressure in mm Hg.

### Analysis

We described characteristics using summary statistics. We compared all biometric outcomes in the immediate intervention group with outcomes in the delayed control group at each follow-up time (i.e. months 1, 2, 3, and 4) using analysis of covariance with the baseline value included as the covariate. These between-group analyses assess the difference between groups at follow-up while controlling for the baseline values. The *P* values <.05 were considered significant. All baseline biometric outcomes (weight, 5% weight loss, waist circumference, and systolic blood pressure) were compared with the outcomes at follow-up visits at months 1, 2, 3, and 4 for within-group analysis for both intervention and control. For within-group analysis, we used paired *t* tests. We used an intention-to-treat analysis. For the outcomes of interest in the present report, data were missing for 8%, 8%, 13%, and 14% at 1, 2, 3, and 4-month follow-up visits respectively. For missing information, we used the last observation carried forward. Supplemental multivariable analyses were performed for each outcome of interest to assess whether sex was a potential moderator of the effectiveness of the intervention. For these models, the explanatory variables included treatment assignment, sex, and the sex-by-treatment interaction effect.

The sample size for this trial was determined for the primary trial end point (step count).^22^ An a priori power analysis was not performed for the secondary outcomes included in this report. In general, for a continuous outcome the sample size used for the current trial provides statistical power (two-tailed, alpha = 0.05) of 80% to detect a difference between groups of 0.5 standard deviations.^22^

## Results

### Participants

Of the 1,587 individuals invited to participate, 244 responded, and 130 of these met inclusion criteria and consented to participate in the study. The full details of the recruitment, enrollment and the CONSORT flow diagram have previously been published. Of 244 potential participants, 130 (53%) consented to participation and were enrolled (overall mean (SD) age, 63.4 (15.0) years). Women comprised 72% of the cohort, and 98% were persons of White non-Hispanic race/ethnicity (Table 1).^22^ The mean (SD) baseline steps, the first 7 days of use, for the immediate intervention group was 5,158 (3,048) steps; for the delayed control group, they were 4,446 (2,422) steps and were not statistically different. We found a statistically significant sex-by-treatment interaction effect (*P* = .03), indicating that the effectiveness of the intervention differed between men and women. Among men, the number of steps was significantly higher in the immediate intervention group than the delayed control group (mean (SD), 6,355 (3,620) vs 3,651 (2,577); *P* = .03); among women, the number of steps was similar between treatment groups (mean (SD), 4,828 (2,702) vs 4,657 (2,361) for immediate intervention vs delayed control; *P* = .99). No differences between the immediate intervention group and the delayed control group existed at baseline with specific attention to sex, cognitive status, and mood.^22^

**Table 1.** Demographics of 130 Participants.

Characteristic	Immediate intervention (*n* = 64)	Control (*n* = 66)
Age (years) mean (SD)	62.2 (SD 17.3)	65.0 (SD 12.2)
Female *n* (%)	43 (67%)	51 (77%)
White race not Hispanic *n* (%)	63 (98%)	64 (97%)

Original data: Takahashi et al.^22^

### Weight

We observed that the mean (SD) baseline weight was 96.0 (18.0) kg in the immediate intervention group and 93.8 (17.6) kg in the delayed control group (Table 2). No difference was found for within-group weight loss in the immediate intervention and delayed control groups at 2- or 4-month follow-up. No patient in either group had ⩾5% weight loss at 2-month follow-up. For both groups, 5% (3 patients each) of the population had a 5% weight loss at 4-month follow-up.

**Table 2.** Weight in Immediate Intervention and Delayed Control Group of 130 Overweight Adults With Multiple Chronic Conditions.

Characteristic	Group		Treatment effect^a^	
	Immediate intervention (*n* = 64)	Delayed control (*n* = 66)	Estimate (95% CI)	*P*
Weight, mean (SD), kg				
Baseline	96.0 (18.0)	93.8 (17.6)		
1 month				
⩾5% weight loss	95.9 (17.8) (*n* = 0 (0%))	94.0 (17.8) (*n* = 0 (0%))	−0.3 (−0.7−0.1)	.20
2 months				
⩾5% weight loss	95.8 (17.8) (*n* = 0 (0%))	93.9 (18.0) (*n* = 0 (0%))	−0.3 (−0.9−0.5)	.44
3 months				
⩾5% weight loss	95.9 (17.8) (*n* = 2 (3%))	93.9 (18.3) (*n* = 2 (3%))	−0.1 (−1.0−0.7)	.98
4 months				
⩾5% weight loss	95.6 (17.7) (*n* = 3 (5%))	93.4 (18.3)^b^ (*n* = 3 (5%))	0.1 (−0.8−1.0)	.86

CI: confidence interval.

a Estimated using analysis of covariance with the baseline value of a given variable included as the covariate.

b *P* < .05 compared with 2 months (comparisons performed only for the delayed group).

### Waist circumference and systolic blood pressure

We observed a significant difference in waist circumference changes between the immediate intervention group and the delayed control group with a net difference, −1.6 cm (95% CI, −3.1 to −0.1) at 2-month follow-up. At baseline, the immediate intervention group had a mean (SD) waist circumference of 115.6 (15.3) cm; the delayed control group had a measurement of 111.8 (14.5) cm (Table 3). At 2 months, mean waist circumference of the immediate intervention group decreased by 0.4 cm while the delayed control group increased by 2.2 cm. No within-group difference in waist circumference was observed for the immediate intervention group. In the delayed control group, we observed a small but significant increase of 2.2 cm in waist circumference from baseline to 2 months (*P* < .05).

**Table 3.** Waist Circumference and Systolic Blood Pressure in Immediate Intervention and Delayed Control Groups in 130 Overweight Adults With Multiple Chronic Conditions.

Variable	Group		Estimate of treatment effect (95% CI)	*P*
	Immediate intervention (*n* = 64) mean (SD)	Delayed control group (*n* = 66) mean (SD)		
Waist circumference, mean (SD), cm				
Baseline	115.6 (15.3)	111.8 (14.5)		
1 months	115.9 (14.8)	113.1 (15.0)	−0.2 (−1.4 to 1.1)	.81
2 months	115.2 (15.4)	114.0 (15.2)^a^	−1.6 (−3.1 to −0.1)	.03
3 months	116.0 (15.1)	113.2 (15.5)	−0.1 (−1.6 to 1.5)	0.94
4 months	115.1 (15.0)	112.9 (15.7)	−0.6 (−2.3 to 1.1)	0.48
Systolic blood pressure, mean (SD), mm Hg				
Baseline	130.0 (15.5)	130.8 (12.9)		
1 months	125.0 (13.2)^a^	129.3 (14.7)	−3.6 (−7.4 to 0.2)	.07
2 months	125.9 (14.7)^a^	128.7 (15.5)	−2.5 (−6.8 to 1.8)	.26
3 months	126.9 (15.3)	131.3 (16.4)	−3.5 (−8.1 to 1.1)	.14
4 months	123.1 (16.1)^a^	128.0 (15.5)	−4.4 (−9.1 to 0.3)	.07

CI: confidence interval.

a *P* < .05 compared with baseline.

No differences were observed between immediate intervention and delayed control group for systolic blood pressure at 2-month follow-up. A decrease in systolic blood pressure was observed at 4 months in the immediate intervention group. Supplemental multivariable analyses were performed for each outcome to assess whether treatment effects differed between males and females. In all cases, no significant treatment-by-sex interaction effects were detected (all *P* > .10). Safety has been previously reported. There were 51 adverse events and 22 serious adverse events including 11 emergency room visits and 11 hospitalizations. After review, these were not identified as secondary to the study.^22^

## Discussion

In this secondary analysis of a randomized controlled trial of SMART goal setting and pedometer use in overweight or obese patients with MCC, we did not observe a decrease in weight between the immediate intervention group and the delayed control group. A small, nonsignificant weight change difference of 0.3 kg between the groups was observed at 2 months which was driven by an increase in waist circumference in the delayed control group. There was no difference in clinical efficacy of >5% weight loss between the two groups.

In other studies, the magnitude of weight change after starting a pedometer program has been modest. In a study of 18 obese women older than 60 years, a significant loss of 4 kg after intensive counseling and pedometer use was observed from baseline to 3 months.^20^ That study had a more intensive counseling component of eight visits with a dietician, as well as a visit with a bariatric physician. Thus, the goal setting and interventions were not comparable with the present study. The lack of efficacy in this study may reflect the lack of an aggressive dietary component. The American Heart Association guidelines emphasize a low-calorie diet as Grade 1 evidence.^20^ The guidelines also recommend 14 visits with a trained interventionist like a dietician over 6 months, which was not performed in this study.^20^ The lack of weight change in the present trial may reflect the lack of effectiveness of the once monthly goal setting, achieving increased step count (as previously reported)^22^ or in changes in dietary behavior of patients who are overweight/obese with MCC. The individuals in this group with MCC may require a more intensive approach to goal setting to achieve improvement in biometric outcomes.^34^ Goal setting in patients with MCC should be more deliberate with shared decision making and longitudinal in nature.^35^

For waist circumference, we observed a within-group increase in waist circumference in the delayed control group compared with baseline (*P* < .05). We observed a significant difference between the immediate intervention group and the delayed control group, with a 1.6-cm change difference in the intervention versus control group at 2 months (*P* = .03) with this increase in waist circumference. We are uncertain why the small increase occurred in waist circumference for the delayed control group, because weight did not change within the group from baseline to 2 months. It is possible that this finding is a result of measurement variability. Waist circumference has less precision than weight or height (i.e. BMI).^36^ One member of the research team did most of the measurements (S.M.Q.); however, other members did measurements also.

Results are mixed about the effect of pedometer programs on waist circumference. In a study of 142 participants using a pedometer with a goal of 10,000 steps daily, the investigators observed a clinically and statistically significant 3.0-cm loss in within-group waist circumference after 6 months in the program.^37^ In other studies, participants with a larger initial waist circumference had the largest amount of change in waist circumference.^38^ Men (N = 299) with >10,000 steps a day had a 3-cm smaller waist circumference than those who had <10,000 steps a day (*P* = .04).^39^ Systolic blood pressure was not significantly different between the intervention and control groups. A significant small within-group decrease of 6.9 mm Hg was found among intervention group from baseline to 4 months. Although the step count increase was not statistically significant as previously reported,^22^ the increase in step count may account for the blood pressure change. In previous cross-sectional studies, increased step count was associated with lower diastolic blood pressure.^40^ In longer term studies using a pedometer among older Asian patients (mean (SD) age, 68.3 (5.8) years), an increased step count decreased the systolic blood pressure.^41^ These findings are encouraging because this occurrence could mitigate heart disease and stroke risk.^42^

Our trial has several limitations. The original study was powered for improvement in step count and not for a weight change. The primary design of the intervention emphasized step count and physical activity, with a secondary emphasis on weight management. Thus, the intervention was not specifically tailored for weight loss. The intervention was designed for a clinical practice that utilized a care coordinator arranging monthly meetings or phone calls with patients. For missing data, we used the last observation carried forward which may introduce bias; however, we had only 8% missing data and we sought a conservative method. Goal setting was at an individual, patient-centered level, rather than a set target of 10,000 steps, to allow individual customization. Recent studies suggest that setting a high step goal may increase step count.^43^ Other trials have emphasized an increase in frequency of goal review sessions.^44,45^ An increase in review frequency may have improved the effectiveness of the intervention in our trial by increasing the opportunity for behavior change. We did not measure adherence with the pedometer use (wear time), which could limit the effectiveness of the intervention. The lack of efficacy of the primary goal of improvement in step count in our previous study^22^ also may have affected our inability to achieve significant weight loss in the immediate intervention group. Finally, the study was conducted with a largely White population which may not generalize to other populations.

### Conclusion

In this secondary analysis of a randomized controlled trial, we did not observe a difference in weight loss between the patient group with SMART goal setting and pedometer use immediately and the group with delayed implementation. These differ from previous studies which show weight loss using goal setting. The primary difference between the studies involves the intensity of the intervention with the current study using a monthly visit with patient-directed goals. These findings may indicate a need for future studies which incorporate tailored, individualized goals and more intensive monitoring to reduce weight in this population of overweight/obese patients with MCC.
